# Histone deacetylase 3-specific inhibitor RGFP966 attenuates oxidative stress and inflammation after traumatic brain injury by activating the Nrf2 pathway

**DOI:** 10.1093/burnst/tkad062

**Published:** 2024-05-04

**Authors:** Lanjuan Xu, Tingting An, Baohui Jia, Qiong Wu, Jinggui Shen, Jie Jin, Jing Liu, Chengjian Li

**Affiliations:** Department of Critical Care Medicine, Zhengzhou Central Hospital affiliated to Zhengzhou University, Zhengzhou, Henan Province 450001, China; Department of Critical Care Medicine, Zhengzhou Central Hospital affiliated to Zhengzhou University, Zhengzhou, Henan Province 450001, China; Department of Critical Care Medicine, Zhengzhou Central Hospital affiliated to Zhengzhou University, Zhengzhou, Henan Province 450001, China; Department of Critical Care Medicine, Zhengzhou Central Hospital affiliated to Zhengzhou University, Zhengzhou, Henan Province 450001, China; Department of Critical Care Medicine, Zhengzhou Central Hospital affiliated to Zhengzhou University, Zhengzhou, Henan Province 450001, China; Department of Critical Care Medicine, Zhengzhou Central Hospital affiliated to Zhengzhou University, Zhengzhou, Henan Province 450001, China; Department of Critical Care Medicine, Zhengzhou Central Hospital affiliated to Zhengzhou University, Zhengzhou, Henan Province 450001, China; Department of Critical Care Medicine, Zhengzhou Central Hospital affiliated to Zhengzhou University, Zhengzhou, Henan Province 450001, China

**Keywords:** TBI, Oxidative stress, Histone deacetylase 3, NRF2, Inflammation

## Abstract

**Background:**

Oxidative stress (OS) and inflammatory reactions play pivotal roles in secondary brain injury after traumatic brain injury (TBI). Histone deacetylase 3 (HDAC3) controls the acetylation of histones and non-histones, which has a significant impact on the central nervous system’s reaction to damage. This research determined the implications of RGFP966, a new and specific inhibitor of HDAC3, for the antioxidant (AO) systems mediated by nuclear factor erythroid2-related factor 2 (Nrf2) and the Nod-like receptor protein 3 (NLRP3) inflammasome in TBI. The study also studied the underlying mechanisms of RGFP966’s actions. Our objective was to examine the impacts and underlying RGFP966 mechanisms in TBI.

**Methods:**

*In vitro*, a rat cortical neuron OS model was induced by H_2_O_2_, followed by the addition of RGFP966 to the culture medium. Neurons were collected after 24 h for western blot (WB), terminal deoxynucleotidyl transferase dUTP nick end labeling (TUNEL) and 2′-7′-dichlorodihydrofluorescein diacetate staining. *In vivo*, RGFP966 (10 mg/kg) was administered post-TBI. Brain tissue water content and modified neurological severity scores were assessed 72 h post-injury. Cortical tissues surrounding the focal injury were subjected to western blot, TUNEL staining, Nissl staining and immunofluorescence/immunohistochemistry staining, and malondialdehyde level, hindered glutathione content and superoxide dismutase activity were measured. Serum was collected for the enzyme-linked immunosorbent assay. Nrf2-specific shRNA lentivirus was injected into the lateral ventricle of rats for 7 days, and cerebral cortex tissue was analyzed by WB and real-time polymerase chain reaction.

**Results:**

During *in vitro* and *in vivo* experiments, RGFP966 suppressed HDAC3 expression, promoted Nrf2 nuclear translocation, activated downstream AO enzymes, mitigated excessive reactive oxygen species production and alleviated nerve cell apoptosis. RGFP966 effectively reduced brain edema and histological damage and enhanced neurological and cognitive function in rats with TBI. RGFP966 markedly inhibited NLRP3 inflammasome activation mediated by high-mobility group box 1 (HMGB1)/toll-like receptor 4 (TLR4). Nrf2 knockdown in TBI rats attenuated the AO and anti-inflammatory, neuroprotective impacts of RGFP966.

**Conclusions:**

Overall, our findings demonstrate that RGFP966 can mitigate the first brain damage and neurological impairments in TBI. The underlying mechanism involves triggering the Nrf2-mediated AO system and negatively regulating the HMGB1/TLR4-mediated NLRP3 inflammasome pathway.

## Graphical Abstract

**Graphical Abstract:**
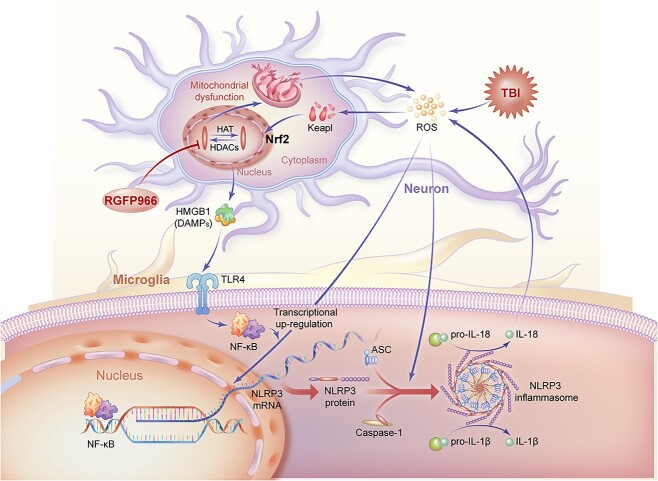

Highlights

- Elevated HDAC3 expression is observed in neurons subjected to H_2_O_2_ treatment and the injured cerebral cortex of TBI rats.
- The specific HDAC3 inhibitor, RGFP966, demonstrates a significant activation of the Nrf2/HO-1/NQO1 antioxidant system. It also inhibits the HMGB1/TLR4 signaling pathway and the formation of inflammatory corpuscles within NLRP3, which leads to a reduction in oxidative stress and neuroinflammation following TBI, consequently decreasing nerve cell apoptosis.
- RGFP966 emerges as a possible candidate for TBI treatment. The Nrf2/HMGB1/TLR4-NLRP3 pathway holds promise as a new therapeutic target for TBI.

## Background

The specific processes responsible for subsequent craniocerebral damage resulting from traumatic brain injury (TBI) remain unclear. Notably, oxidative stress (OS) and inflammatory responses are acknowledged as pivotal pathobiological characteristics of secondary brain damage, contributing to additional damage such as edema and nerve cell death [1, 2]. Neurons, being highly susceptible to OS, can sustain indirect damage to their DNA, lipids and proteins stimulated by reactive oxygen species (ROS). These ROS can also trigger various signal transduction pathways in neurons [3]. Damaged neurons release damage-associated molecular patterns encompassing ATP, RNA and high-mobility group box 1 (HMGB1), promoting the activation of inflammatory cells like microglia and significantly elevating levels of inflammatory mediators, encompassing cytokines and chemokines [4]. Excessive proinflammatory factors further intensify OS, heighten mitochondrial dysfunction and facilitate neuronal apoptosis [5]. Data from animal studies have consistently indicated that an elevated antioxidant (AO) response and reduced inflammation contribute to mitigating brain damage [6, 7].

Nuclear factor erythroid 2 related factor 2 (Nrf2) is vital as a transcription factor and primary controller in cellular reactions to OS. Following stimulation, Nrf2 moves into the nucleus and attaches to the antioxidant response element (ARE) located in the AO genes’ promoter region. This stimulation leads to the production of AO proteins downstream, encompassing NAD(P)H quinone oxidoreductase-1 (NQO-1), heme oxygenase-1 (HO-1), glutathione-S-transferase, superoxide dismutase (SOD) and glutathione peroxidase [8]. The Nrf2-dependent gene products persist in safeguarding cells from oxidation or harm caused by external substances. The Nrf2-ARE pathway is acknowledged for its defensive function in several disorders of the central nervous system [9].

HMGB1, a member of damage-associated molecular patterns, functions as a proinflammatory cytokine-like factor [10]. HMGB1 binds to toll-like receptors (TLRs) on microglia, following its secretion by inflammatory cells or release from injured neurons into the extracellular space. This interaction triggers the activation and movement of nuclear factor-κB (NF-κB) into the nucleus. Subsequently, it attaches to particular DNA sequences, enhancing the gene transcription responsible for inflammatory cytokines [11].

The NLRP3 inflammasome is a multi-protein complex composed of the NLRP3 scaffold, cysteine-requiring aspartate protease1 (Caspase1) and apoptotic speck-containing protein (ASC) adaptor [12]. In response to trauma, it is established that ROS [13], HMGB1 [14] and heat shock proteins [15] can activate the NLRP3 inflammasome through TLRs and NF-κB signals, which results in the maturation and secretion of proinflammatory cytokines, encompassing interleukin (IL)-1β and IL-18 [12]. Both IL-1β and IL-18 contribute to the accumulation of ROS, creating a detrimental feedback loop between OS and NLRP3 activation [16].

Histone deacetylases (HDACs) are a group of enzymes that facilitate the histone deacetylation and the regulation of more than 50 types of non-histone proteins [17]. Histones undergo acetylation post-translation, providing epigenetic control over target gene transcription [18]. Numerous papers have demonstrated the link between HDAC3 with OS and inflammatory damage [19, 20]. RGFP966, a very effective inhibitor of HDAC3, was recently illustrated to have AO and anti-inflammatory properties via stimulating the Nrf2 signaling pathway in disorders outside of the central nervous system [21, 22]. Nevertheless, it is still unclear whether RGFP966 has comparable protective benefits in TBI. Hence, we performed research to examine the influence of RGFP966 on the initial brain damage in a rat model of OS injury in cerebral cortex neurons and a rat model of TBI. Our objective was to clarify the underlying molecular pathways involved.

## Methods

### Animals

Sprague–Dawley rats at 18 days of pregnancy and adult male SD rats (weighing 220 ± 20 g) were obtained from the Animal Research Institute of Zhengzhou University. Before the experiment, they were kept in an environment with clear day and night cycles and relatively constant temperature and humidity for 7 days, and had *ad libitum* availability of food and water.

### Experiment design

#### Experiment 1

In all, 8 pregnant rats and 53 fetal rats were utilized for *in vitro* trials. Primary cortical neurons were allocated into four groups in a random manner: control, TBI, TBI + vehicle [dimethyl sulfoxide (DMSO)] and TBI + RGFP966. Neuronal morphology post-OS injury was observed under an optical microscope. Evaluation of the severity of injury included measurements of malondialdehyde (MDA), lactate dehydrogenase and cell activity in the supernatant. All groups underwent western blot (WB) analysis, immunofluorescence (IF) staining, TdT-mediated dUTP nick end labeling (TUNEL) staining and 2′-7′-dichlorodihydrofluorescein diacetate (DCFH-DA) staining.

#### Experiment 2

A total of 72 rats were included in the experiment (80 rats survived post-operation, and 72 rats were eligible). The rats were allocated to four groups using random allocation: sham, TBI, TBI + vehicle and TBI + RGFP966. Modified neurological severity scores (mNSS) and brain water content were measured 72 h post-trauma.

WB, MDA level, reduced glutathione (GSH) content and SOD activity were measured in cortex brain tissue of rats. Paraffin sections were obtained for TUNEL, hematoxylin–eosin (HE), Nissl and IF staining. Serum was gathered for the enzyme-linked immunosorbent assay (ELISA).

#### Experiment 3

Initially, 12 rats were separated into four groups in a random manner: sham, TBI, TBI + non coding-short hairpin RNA (NC-shRNA) and TBI + Nrf2-shRNA (n = 3 per group). They were euthanized 72 h post-TBI to confirm the Nrf2-specific shRNA efficacy by WB and quantitative real-time polymerase chain reaction (RT-PCR). Subsequently, 15 rats were randomly assigned into five groups: sham, TBI, TBI + RGFP966, TBI + RGFP966 + NC-shRNA and TBI + RGFP966 + Nrf2-shRNA for WB (n = 3 per group).

### Primary cortical neuron culture

After removing the brain from fetal rats, the cerebral cortex tissue was carefully separated and cut. Ethylene Diamine Tetraacetic Acid (EDTA)-0.05% trypsin (SolarBio, Beijing) was added for digestion at 37°C for 30 min. Digestion was stopped by adding 20% Fetal Bovine Serum-Dulbecco’s Modified Eagle Medium (FBS-DMEM) complete medium (Procell, Wuhan). The cells were passed through a 70 μm cell sieve, centrifuged at 1500 rpm for 5 min and resuspended in complete medium. The cells were placed in coated bottles and the medium was changed every 3 days. On day 7, 5 μg/ml cytarabine (Solar Bio, Beijing) was added for 72 h to inhibit cell proliferation other than neurons. Mature neurons were then utilized for *in vitro* trials.

### Establishment of *in vitro* and *in vivo* models

To simulate TBI *in vitro*, a H_2_O_2_-induced neuronal OS model was employed. Neurons were treated with 1 mM H_2_O_2_, diluted to an ultimate concentration of 150 μM, and analyzed 24 h post-treatment.

For the *in vivo* model, a stable rat hydraulic percussion brain injury model was established using standardized surgical techniques and international hydraulic percussion instruments. Rats were administered intraperitoneal chloral hydrate (0.3 ml/10 g) to induce anesthesia and subjected to a longitudinal median incision to expose the skull. A 4 mm bone window was drilled using an electric bone drill centering at coordinates 1.80 mm to the right and 1.5 mm posterior to the anterior fontanelle, ensuring the integrity of the dura mater. The right hammer was released to impact the brain with normal saline, creating a controlled hydraulic injury. Subsequently, the bone window was sealed with bone wax, the scalp was stitched and the rats were returned to their cages. Only scalp incision and bone window drilling were performed without impact in the sham group.

### Drug administration

In the *in vitro* experiment, RGFP966 was dissolved in DMSO stock solution, diluted to 15 μM with serum-free medium [23] and added 1 h after neuronal injury. In the *in vivo* experiment, RGFP966 (10 mg/kg) [24] was dissolved in a 1% DMSO stock solution and administered intraperitoneally twice daily immediately after TBI. Control groups received an equal dose of DMSO.

### RNA interference

In brief, the heads of anesthetized rats were secured in the brain stereotactic apparatus. A hole with a diameter of 1 mm was bored, starting at the anterior fontanelle, 1.8 mm to the right and 1.5 mm to the back, targeting the right lateral ventricle. An 8 μl solution of Nrf2-shRNA or NC-shRNA lentivirus (Genepharma, Shanghai) was drawn into a micro-syringe, which was then slowly and vertically inserted into the drilling point at a depth of 3.5 mm. The solution was injected at a rate of 0.5 μl/min. After injection, the needle was slowly withdrawn. Rats in the control group underwent skull drilling and an equivalent dose of normal saline was injected into the intracerebroventricular space. Experimental TBI was induced 7 days after intracerebroventricular injection. The Nrf2-shRNA sequence was 5′CCCTGTTGATGACTTCAATGA-3′, with a non-targeting RNA sequence acting as a negative control.

### WB

The extraction of total proteins was conducted from neurons and tissues around the diseased cortex in rats, and the protein concentration was measured using a Bicinchoninic acid (BCA) protein quantitative kit (Epizyme, Shanghai). The protein samples were evenly divided and subjected to polyacrylamide gel electrophoresis. Subsequently, they were placed onto polyvinylidene fluoride (PVDF) membranes and subsequently treated with 5% skim milk to prevent non-specific binding for 2 h at ambient temperature. The PVDF membranes were subjected to incubation overnight at 4°C with primary antibodies (Abs) targeting HDAC3, H3, H4, acetyl-H3, acetyl-H4 (Abcam, UK), Nrf2, HO-1, Neuron Specific Enolase (NSE), Caspase1, IL-1β, HMGB1, TLR4, BCL2-Associated X (BAX), B-cell lymphoma-2 (BCL-2), β-actin (Proteintech, Wuhan), NQO1 (Bioworld, UK) and Ionized calcium bindingadaptor molecule-1 (Iba-1)(Cell Signaling Technology, USA). The PVDF membranes were then treated with the matching secondary Abs linked to Horseradish Peroxidase (HRP) (Epizyme, Shanghai) for 1 h at ambient temperature on the next day. Protein bands were visualized by employing enhanced chemiluminescence (Epizyme, Shanghai), and the optical density of protein strips was measured using Image J software.

### DCFH-DA staining

Neurons in each group were incubated with a 1 : 1000 diluted DCFH-DA probe (Beyotime, Shanghai) at 37°C for 1 h. Following three rinses with PBS, the cells were promptly captured using an inverted fluorescent microscope. Image J software was employed to analyze the mean fluorescence intensity.

### Neurological function testing

Researchers who were unaware of the experiment conducted the mNSS on rats 72 h after a TBI. The mNSS comprises assessments of motor, sensory, balance and reflex functions. Neurological function is evaluated on a scale ranging from 0 to 18 points, with scores of 1 to 6 revealing mild injury, scores of 7 to 12 revealing moderate injury, and scores of 13 to 18 revealing severe injury.

### Measurement of brain water content

Brain water content (BWC) was assessed using a wet/dry weight technique. The damaged cerebral cortex was promptly removed and the weight of the moist sample was documented. Afterward, the brain tissue was desiccated in an oven at 100°C for 48 h and the weight of the tissue after dehydration was measured. BWC was determined by applying the formula [(wet weight − dry weight)]/(wet weight) × 100%.

### IF staining

Neurons were cultivated on ascending surfaces and fixed using a 4% paraformaldehyde solution. The damaged cortical tissues of rats were fixed, encased in paraffin for preservation and sliced into sections with a 4 μm thickness. Following the removal of wax and the recovery of antigens using citric acid, the sections were then sealed with 5% BSA for 30 min at ambient temperature. The main Abs used for overnight incubation were HDAC3 (1:300, Abcam), Nrf2 (1:250, Proteintech), NSE (1:100, Proteintech) and Iba-1 (1:200, Cell Signaling Technology). Following the washing step, the sections were exposed to the secondary Ab for 1 h. The nuclei were stained with 4′,6-diamidino-2-phenylindole (DAPI) and images were captured using an inverted fluorescence microscope. The mean fluorescence intensity was assessed by employing Image J software.

### Immunohistochemistry staining

Following antigen retrieval, slices with a thickness of 4 μm were subjected to overnight incubation at 4°C with anti-NQO1 (1:50, Bioworld) and anti-HO-1 (1:100, Proteintech) Abs. Following the washing step, the sections were exposed to a secondary Ab at ambient temperature for 30 min. The presence of immune response was identified using 3,3-diaminobenzidine and then the sample was stained with hematoxylin for contrast. The pictures were obtained via the use of a microscope and analyzed employing Image J software.

### TUNEL staining

The cells on climbing plates or brain slices were exposed to incubation with a TUNEL reaction mixture (Solarbio, Beijing) and kept at a temperature of 37°C for 1 h. Following the PBS wash, the slides were exposed to DAPI for 3 min to facilitate nuclear staining. Subsequently, the slides were promptly captured using an inverted fluorescent microscope. The TUNEL-positive cells were measured employing ImageJ software and the ratio of cells undergoing apoptosis to cells labeled with DAPI was determined as the apoptosis index (apoptotic cells/DAPI).

### Nissl staining

After dewaxing, the staining of paraffin sections was conducted with a Nissl dyeing solution (Solarbio, Beijing) for 5 min. After washing and alcohol dehydration, xylene was used for transparency for 5 min, and neutral gum was applied for sealing. Images were captured under an optical microscope.

### HE staining

The paraffin slices underwent dewaxing and were thereafter stained with hematoxylin for 5 min. The process of differentiation was carried out using a solution of 1% hydrochloric acid in alcohol for 30 s, followed by soaking in clear water for 15 min. Eosin was used for staining for 30 s, and sections were dehydrated, made transparent and sealed. Images were captured under an optical microscope.

### MDA, GSH and SOD quantification

After brain homogenization, the measurement of MDA, GSH and SOD levels was performed based on the guidelines of the MDA detection kit (Solarbio, Beijing), GSH detection kit (Nanjing Jiancheng, Nanjing) and SOD activity detection kit (Nanjing Jiancheng, Nanjing).

### ELISA assay

Serum inflammatory cytokines were distinguished by ELISA kits for Tumor Necrosis Factor (TNF)-α (Cloud-Clone Corp, Wuhan), IL-1β (Solarbio, Beijing) and IL-18 (Solarbio, Beijing), following the manufacturer’s instructions.

### RT-PCR

The Trizol reagent (Beyotime, Shanghai) was used to isolate total mRNA from tissues, which was then quantified employing spectrophotometric analysis (OD260/OD280). The mRNA was converted into cDNA through reverse transcription using a cDNA synthesis kit from Sangon Biotech, Shanghai. Subsequently, PCR amplification was carried out using the UltraSYBR Mixture kit from CWBIO, Shanghai. The Nrf2 forward and reverse primers were 5′-TGCTCCGACTAGCCATTGAC-3′ and 3′-TGTCAATCAAATCCATGTCCTGC-5′.

### Statistical analysis

Data are presented as mean ± standard deviation. Data analysis and visualization were performed employing GraphPad Prism 9.0, developed by GraphPad Software Inc., CA, USA. The *t*-test was utilized to compare two groups at a certain time point, whereas one-way analysis of variance and the least significant difference test was employed to compare several groups. Statistical significance was determined at a significance level of ^*^p < 0.05 and ^**^p < 0.01.

## Results

### Determination of neurons in rat cerebral cortex and preparation of OS model *in vitro*

**Figure 1:**
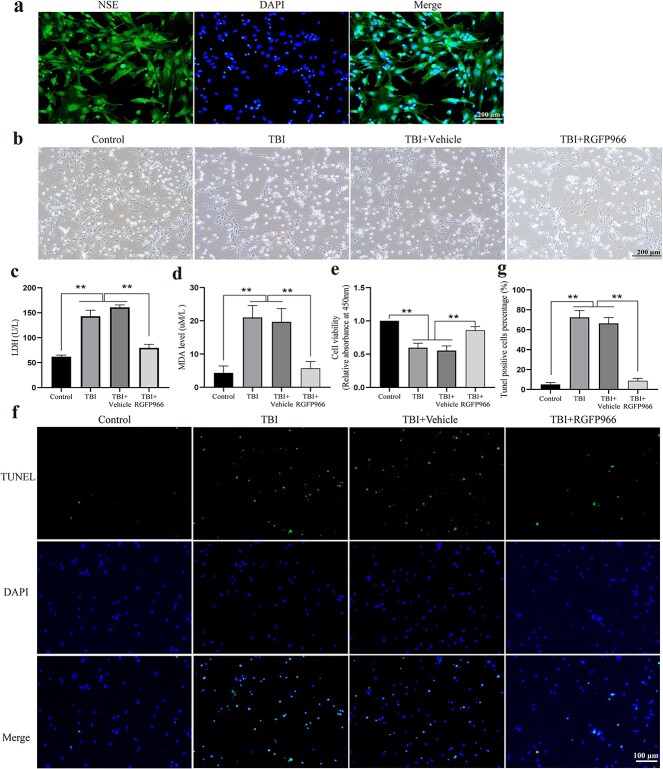
Identification of primary neurons in the rat cerebral cortex and the effect of RGFP966 on TBI primary neurons. (**a**) Morphology of primary neurons under a light microscope. Scale bar: 200 μm. (**b**) A representative microscopic picture of NSE immunofluorescence staining of primary neurons. Scale bar: 200 μm. (**c**) LDH content in the supernatant of each group. n = 3 per group. (**d**) MDA content in the supernatant of each group. n = 3 per group. (**e**) Cell viability in each group. n = 3 per group. (**f**) Representative TUNEL staining photomicrographs in the pericontusional cortex. Scale bar: 100 μm. n = 3 per group. (**g**) TUNEL positive cell percentage. ^*^^*^p < 0.01. *NSE* neuron-specific enolase, *TBI* traumatic brain injury, *TUNEL* TdT-mediated dUTP nick end labeling, *LDH* lactate dehydrogenase, *MDA* Malondialdehyde

Neuron cells in the rat cerebral cortex were identified through NSE staining using cellular IF (Figure 1a). Most cells expressed NSE, a marker of neurons, confirming that the cultured cells were indeed neurons. In order to evaluate the AO properties of RGFP966, H_2_O_2_ was used to stimulate primary neurons, creating an *in vitro* model of OS. Under the optical microscope, increased cell debris and a reduction in neuronal cell bodies and processes were observed after H_2_O_2_ stimulation (Figure 1b).

### RGFP966 improves H_2_O_2_-induced injury of primary neurons

As depicted in Figure 1c and d, RGFP966 significantly reduced the lactate dehydrogenase and MDA levels in the supernatant of neurons stimulated by H_2_O_2_ (*p* < 0.01). Additionally, RGFP966 significantly improved cell viability (*p* < 0.01) (Figure 1e). TUNEL staining revealed a high number of apoptotic neurons after H_2_O_2_ stimulation (*p* < 0.01), and RGFP966 effectively attenuated the presence of apoptotic positive cells following H_2_O_2_ stimulation (*p* < 0.01) (Figure 1f and g).

### RGFP966 inhibits the expression of HDAC3 in primary neurons induced by H_2_O_2_, stimulates the Nrf2/HO-1/NQO1 signaling pathway and reduces the production of ROS

**Figure 2:**
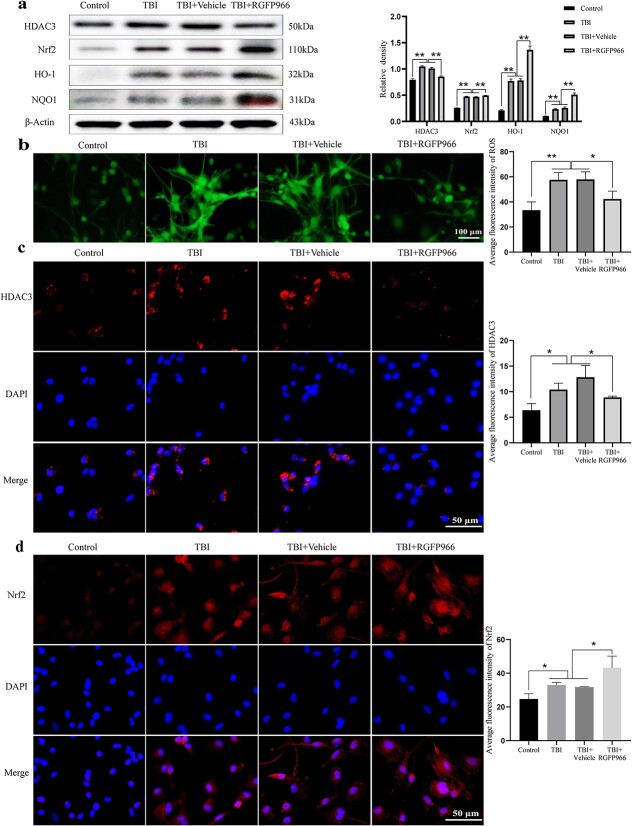
Effect of RGFP966 on oxidative stress of TBI primary neurons. (**a**) Western blot bands of HDAC3, Nrf2, HO-1, and NQO1 in each group and quantification of relative protein expression. n = 3 per group. (**b**) The representative fluorescence micrograph of DCFH-DA staining of primary neurons in each group. Scale bar: 100 μm. n = 3 per group. (**c**) The representative microscopic pictures of cell immunofluorescence of primary neuron cells HDAC3 in each group and quantification. Scale bar: 50 μm. n = 3 per group. (**d**) The representative microscopic picture of cell immunofluorescence of primary neuron Nrf2 in each group and quantification. Scale bar: 50 μm. n = 3 per group. ^*^p < 0.05, ^*^^*^p < 0.01. *TBI* traumatic brain injury, *HDAC3* histone deacetylase 3, *Nrf2* nuclear factor erythroid 2 related factor 2, *HO-1* heme oxygenase-1, *NQO1* NAD(P)H quinone oxidoreductase-1

WB indicated that RGFP966 effectively decreased the protein levels of HDAC3 in neurons that were activated by H_2_O_2_ while significantly increasing the protein expressions of Nrf2, HO-1 and NQO1 (Figure 2a). The ROS content of primary neurons in each group, determined by DCFH-DA staining (Figure 2b), showed that H_2_O_2_ significantly elevated the ROS level compared with the control group (*p* < 0.01), and the reversion of this effect was achieved with RGFP966 treatment (*p* < 0.05). The outcomes of IF staining were aligned with those of WB: i.e., under oxidative damage conditions, after RGFP966 treatment, the total red fluorescence intensity of HDAC3 in cells was significantly weakened (*p* < 0.05) (Figure 2c), while the total red fluorescence intensity of Nrf2 in cells was more intense, especially in the nucleus (*p* < 0.05) (Figure 2d).

### Effects of RGFP966 treatment on neurological function, brain edema and histological damage after TBI

**Figure 3:**
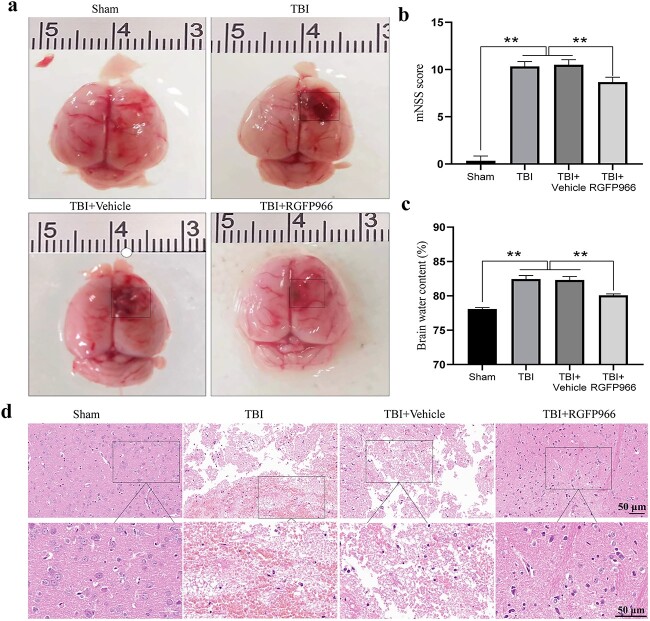
Effects of RGFP966 treatment on neurological function, brain edema, and histological damage after TBI. (**a**) Typical macroscopic pictures of rat brains in each group. (**b**) Statistical chart of mNSS of rats in each group. n = 6 per group. (**c**) Measurement of BWC in the cerebral cortex of rats in each group. n = 6 per group. (**d**) HE staining was used to evaluate the histology of the cerebral cortex around the hematoma. Scale bar: 50 μm. n = 3 per group. ^*^p < 0.05, ^*^^*^p < 0.01. *TBI* traumatic brain injury, *mNSS* modified neurological severity scores, *BWC* brain water content, *HE* hematoxylin–eosin

Figure 3a displays a representative macroscopic view of rat brains. The mNSS evaluation of neurological function (Figure 3b) revealed that the mNSS score of the TBI group exhibited a significant increase compared with the sham group (*p* < 0.01), indicating substantial neurobehavioral defects. The mNSS score decreased significantly after RGFP966 administration (*p* < 0.01 *vs* TBI + vehicle). BWC increased significantly 72 h after TBI (*p* < 0.01 *vs* sham) and RGFP966 treatment hindered brain water content following TBI (*p* < 0.01 *vs* TBI + vehicle) (Figure 3c). Histological assessment using HE staining showed that RGFP966 treatment significantly improved histological damage around the hematoma (Figure 3d).

### RGFP966 reduced the apoptosis and neuron loss caused by TBI

**Figure 4:**
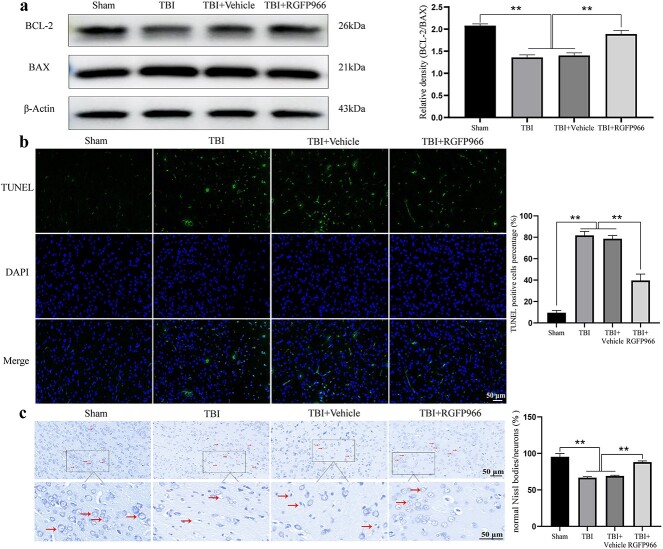
RGFP966 reduces apoptosis and neuron loss caused by TBI. (**a**) Western blot bands of BAX and BCL-2 and quantification of relative protein expression. n = 3 per group. (**b**) The representative fluorescence micrographs of TUNEL staining and quantification. Scale bar: 50 μm. n = 3 per group. (**c**) Nissl staining pictures and quantification. Scale bar: 50 μm. n = 3 per group. ^*^p < 0.05, ^*^^*^p < 0.01. *TBI* traumatic brain injury, *BCL-2* B-cell lymphoma/leukemia 2, *BAX* BCL2-Associated X, *TUNEL* TdT-mediated dUTP nick end labeling

Apoptosis-related protein expression was assessed by WB (Figure 4a). The results showed that RGFP966 treatment could increase the BCL-2/BAX ratio in the diseased cortex of rats (*p <* 0.01). TUNEL staining indicated that RGFP966 treatment significantly hindered the number of apoptosis-positive cells following TBI (Figure 4b). The above data substantiated that RGFP966 could reduce the apoptosis of nerve cells in TBI rats (*p* < 0.01). Then, we used Nissl staining to observe the basic structure of cortical neurons in each group of rats (Figure 4c). The results showed that the neurons in the cerebral cortex of rats in the sham group were clear and complete, with large nuclei and obvious nucleoli, and Nissl bodies, in dark blue, were large and numerous. In contrast, Nissl bodies in the cytoplasm of injured neurons in the TBI group were condensed and necrotic, and vacuole-like structures were formed after dissolution and liquefaction (*p <* 0.01). After RGFP966 treatment, the degree of neuron injury was obviously reduced (*p <* 0.01). The findings suggest that therapy with RGFP966 has a neuroprotective impact on the damaged cortex following TBI.

### RGFP966 inhibited HDAC3 expression and increased histone acetylation in TBI

**Figure 5:**
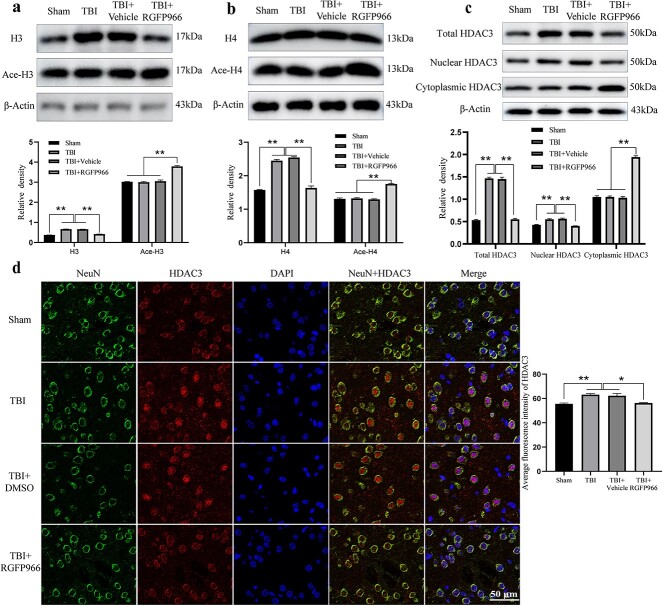
RGFP966 inhibits HDAC3 expression and increases histone acetylation in lesioned cortices. (**a**) Western blot bands of H3 and acetylated H3 and quantification of relative protein expression. n = 3 per group. (**b**) Western blot bands of H4 and acetylated H4 and quantification of relative protein expression. n = 3 per group. (**c**) Western blot bands of nucleus, cytoplasm, and total HDAC3 protein and quantification of relative protein expression. n = 3 per group. (**d**) Immunofluorescence chemical representative microscope pictures of HDAC3 and quantification. Scale bar: 50 μm. n = 3 per group. ^*^p < 0.05, ^*^^*^p < 0.01. *TBI* traumatic brain injury, *HDAC3 *histone deacetylase 3, *DAPI *4′,6-diamidino-2-phenylindole, *NeuN* neuronal nuclei

WB analysis demonstrated that RGFP966 significantly elevated the acetylation levels of histone H3 and H4 during TBI (*p* < 0.01) (Figure 5a and b). The expression of nuclear and total HDAC3 protein in the TBI group was significantly elevated (*p* < 0.01). After RGFP966 treatment, HDAC3 expression in the nucleus and total protein decreased significantly (*p* < 0.01), while HDAC3 expression in the cytoplasm was elevated significantly (*p* < 0.01) (Figure 5c). IF further confirmed that RGFP966 decreased HDAC3 expression in the lesioned cortex (*p* < 0.05) (Figure 5d). The outcomes suggest that RGFP966 has the potential to protect neurons from apoptosis caused by TBI by suppressing HDAC3 expression.

### RGFP966 increased AO factors through Nrf2/HO-1 signaling pathway activation

**Figure 6:**
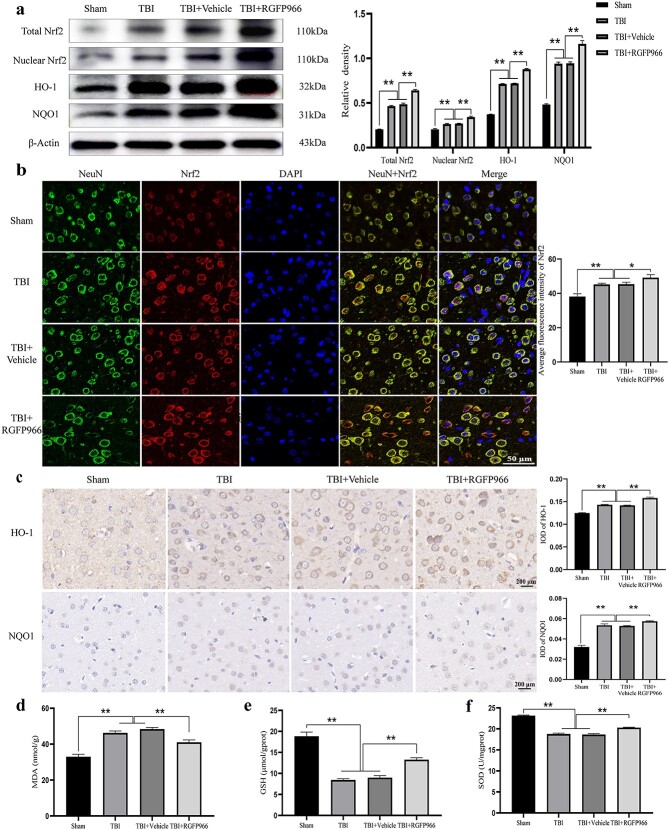
RGFP966 increases antioxidative factors through activation of the Nrf2 signaling pathway. (**a**) Western blot bands of the nucleus, total Nrf2, HO-1, and NQO1, and quantification of relative protein expression. n = 3 per group. (**b**) Immunofluorescence chemical representative microscope pictures of Nrf2 and quantification. Scale bar: 50 μm. n = 3 per group. (**c**) Immunohistochemical microscopic pictures of HO-1 and NQO1 and quantification. Scale bar: 20 μm. n = 3 per group. (**d**) MDA, GSH, and SOD levels in the cerebral cortex of rats in each group. n = 6 per group. ^*^p < 0.05, ^*^^*^p < 0.01. *TBI* traumatic brain injury, *Nrf2* nuclear factor erythroid2 related factor 2, *HO-1* heme oxygenase-1, *NQO-1* NAD(P)H quinone oxidoreductase-1, *MDA* malondialdehyde, *GSH* glutathione, *SOD* superoxide dismutase, *NeuN* neuronal nuclei

WB showed that the expression of total Nrf2 and nuclear Nrf2 in the TBI group was significantly greater than in the sham group (*p* < 0.01). Total Nrf2 and nuclear Nrf2 expression in TBI rats treated with RGFP966 was further increased (*p* < 0.01) (Figure 6a). Immunostaining confirmed that treatment with RGFP966 resulted in an elevation in the overall amount of Nrf2 protein and facilitated the movement of Nrf2 into the nucleus in neurons (Figure 6b). The immunohistochemistry results were in agreement with the WB findings, indicating that the administration of RGFP966 led to a significant upregulation of the protein expression of HO-1 and NQO1 in the affected cortex (*p* < 0.01). RGFP966 treatment reduced the MDA level in the lesioned cortex while significantly increasing SOD and GSH levels (*p* < 0.01) (Figure 6d-f).

### RGFP966 inhibits the inflammatory response mediated by microglia in the lesioned cortex following TBI

**Figure 7:**
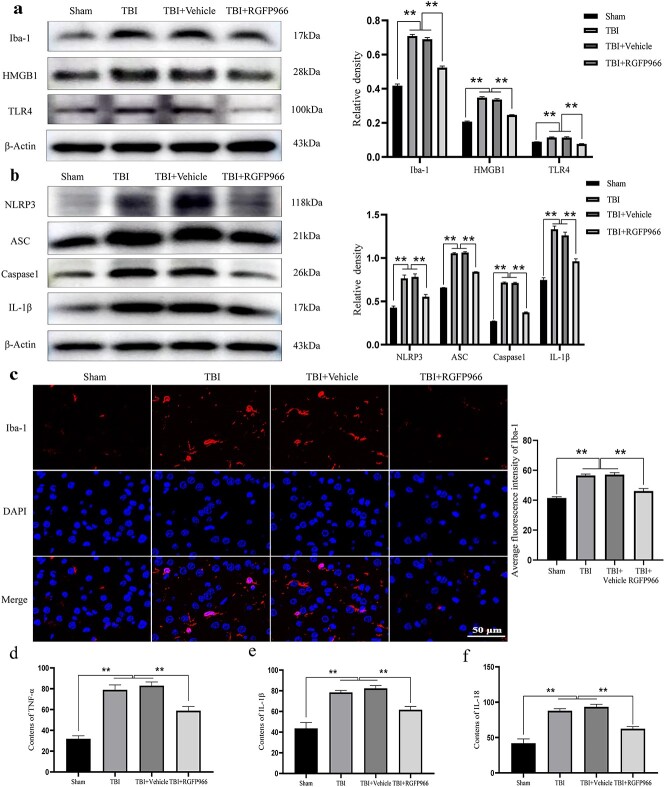
Effect of RGFP966 treatment on activation and inflammatory response of microglia in the diseased cortex. (**a**) Western blot bands of Iba-1, HMGB1, and TLR4 in the diseased cortex of rats in each group and quantification of relative protein expression. n = 3 per group. (**b**) Western blot bands of NLRP3, ASC, Caspase1, and IL-1β in the lesioned cortex of rats in each group and quantification of relative protein expression. n = 3 per group. n = 3 per group. (**c**) Immunofluorescence chemical representative microscope pictures of Iba-1 in the lesioned cortex of rats in each group and quantitative charts. Scale bar: 50 μm. n = 3 per group. The contents of (**d**) TNF-α, (**e**) IL-1β, and (**f**) IL-18 in the lesioned cortex of rats. n = 6 per group. ^*^^*^p < 0.01. *TBI* traumatic brain injury, *Iba-1* ionized calcium-binding adapter molecule 1, *TNF-α* tumor necrosis factor-α, *IL* interleukin

Considering the crucial role of microglia activation and resulting neuroinflammatory reaction in secondary injury following TBI, WB (Figure 7a) and IF (Figure 7c) indicated that activated microglia (Iba-1 positive) increased in the cortex around the lesion following TBI (*p* < 0.01). Furthermore, RGFP966 treatment significantly suppressed microglial activation (*p* < 0.01). WB outcomes consistently showed that the contents of HMGB1 and TLR4 in the lesioned cortex of rats treated with RGFP966 were significantly reduced compared with TBI rats (*p* < 0.01).

In addition, RGFP966 suppressed the expression of NLRP3 inflammasome components and the subsequent release of IL-1β/IL-18. WB demonstrated that NLRP3, ASC, Caspase1 and IL-1β protein levels in the lesioned cortex of TBI rats were significantly higher than those in the sham group (*p* < 0.01). Nevertheless, the protein levels of NLRP3, ASC, Caspase1 and IL-1β decreased significantly after RGFP966 treatment (Figure 7b) (*p* < 0.01). ELISA showed that the levels of pro-inflammatory cytokines (TNF-α, IL-1β and IL-18) in the serum of rats treated with RGFP966 were significantly reduced compared with those of rats with TBI (Figure 7d–f) (*p* < 0.01).

### Interfering with Nrf2 diminished the AO and anti-inflammatory properties of RGFP966 in treating TBI

**Figure 8:**
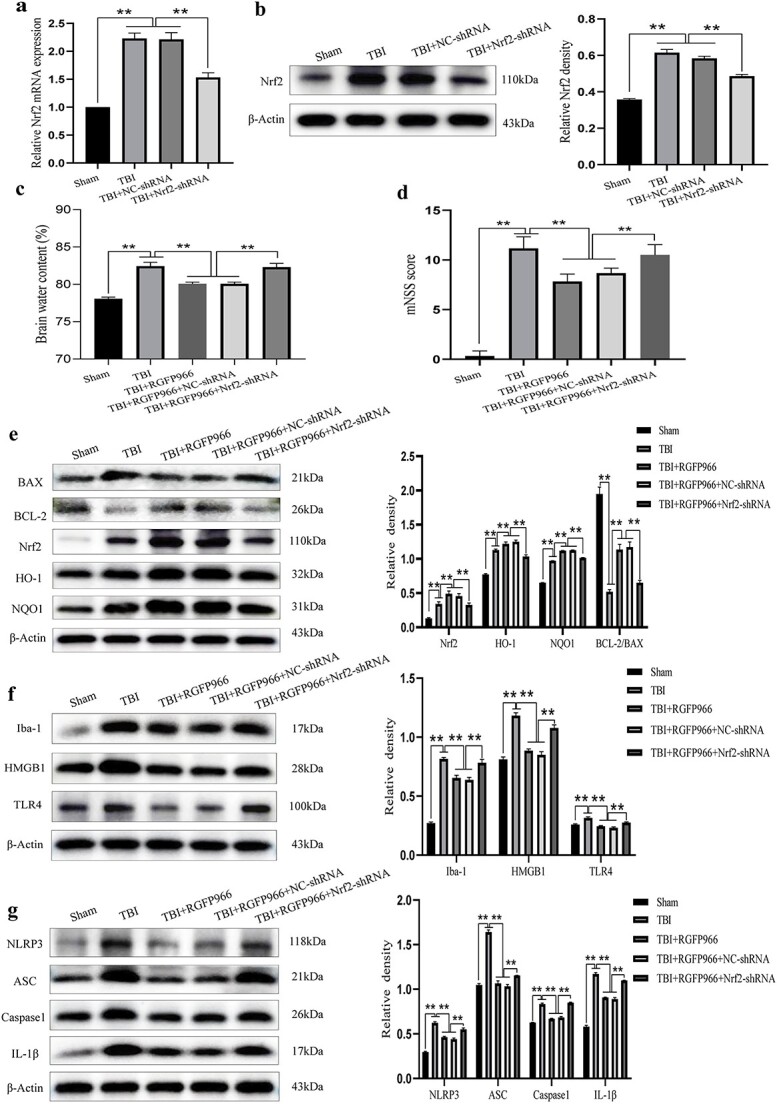
Nrf2 interference weakened the antioxidant and anti-inflammatory effects of RGFP966 on TBI. (**a**) RT-PCR results of Nrf2. (**b**) Western blot bands of Nrf2 and quantification of relative protein expression. (**c**) Brain edema content in each group. n = 3 per group. (**d**) mNSS score of rats in each group. (**e**) Western blot bands of Nrf2, HO-1, NQO1, BAX, and BCL-2 and quantification of relative protein expression. n = 6 per group. (**f**) Western blot bands of Iba-1, HMGB1, and TLR4 and quantification of relative protein expression. n = 3 per group. (**g**) Western blot bands of NLRP3, ASC, Caspase1, and IL-1β and quantification of relative protein expression. n = 3 per group. ^*^p < 0.05，^*^^*^p < 0.01. *TBI* traumatic brain injury, *Nrf2* nuclear factor erythroid2 related factor 2, *mNSS* modified neurological severity scores, *IL* interleukin, *NQO1* NAD(P)H quinone oxidoreductase-1, *BAX* BCL2-Associated X, *BCL* B-cell lymphoma, *HO-1* heme oxygenase-1, *ASC* apoptotic speck-containing protein

To investigate whether the neuroprotective actions of RGFP966 post-TBI rely on Nrf2 activation, we co-administered RGFP966 with Nrf2-shRNA lentivirus. The results revealed a significant inhibition of Nrf2 expression at both mRNA and protein levels with Nrf2-shRNA lentivirus (Figure 8a and b).

The downregulation of Nrf2 compromised the positive effects of RGFP966 on TBI prognosis recovery. RGFP966 treatment noticeably alleviated brain edema and promoted early motor function recovery post-TBI. However, upon Nrf2 knockdown by lentivirus, this alleviation was attenuated, indicating that the downregulation of Nrf2 hindered the therapeutic effect of RGFP966 (Figure 8c and d).

WB results demonstrated a significant decrease (*p* < 0.01) in the protein levels of Nrf2, HO-1 and NQO1 in the TBI + RGFP966 + Nrf2-shRNA group compared to the TBI + RGFP966 + NC-shRNA group. Moreover, there was a significant elevation in BAX protein content and a reduction in BCL-2 protein content (*p* < 0.01) (Figure 8e).

Regarding microglia activation and HMGB1/TLR4, the protein levels of Iba-1, HMGB1 and TLR4 significantly increased after Nrf2-shRNA lentivirus interference (*p* < 0.01 *vs* TBI + RGFP966 + NC-shRNA) (Figure 8f).

Finally, the expression of NLRP3 inflammasome components was assessed. Results showed that Nrf2 knockdown significantly elevated the levels of NLRP3, ASC, Caspase1 and IL-1β protein expression compared with the TBI + RGFP966 + NC-shRNA group (*p* < 0.01) (Figure 8g).

These findings indicate that at the molecular level, RGFP966 could inhibit microglia activation and the NLRP3 inflammasome pathway by promoting the AO pathway mediated by Nrf2 activation. The silencing of Nrf2 by Nrf2-shRNA lentivirus mitigated the beneficial effect of RGFP966.

## Discussion

It is now understood that the secondary injury of TBI involves a complex pathophysiological process encompassing OS, mitochondrial dysfunction, microglia activation and inflammatory reactions, all contributing to neuronal apoptosis and impacting neural function [25]. HDAC3, a class I HDAC widely expressed in the brain, regulates histone and protein acetylation levels [26]. Previous studies indicate that HDAC3 inhibition may enhance neuronal survival under preconditioning conditions [27]. Despite the promising aspects, nonspecific HDAC inhibitors faced dose-limiting toxicity issues in clinical trials [28]. RGFP966, a highly selective HDAC3 inhibitor, has demonstrated neuroinhibitory and neuroprotective impacts in several central nervous system diseases, encompassing neurodegenerative diseases [29], cerebral ischemia [30] and spinal cord injury [31]. While RGFP966 efficiently penetrates the blood–brain barrier with high specificity and minimal side effects, its function in TBI is yet uncertain. In our research, we explored the AO effects of RGFP966 on primary neurons, revealing its ability to inhibit HDAC3 expression, decrease ROS production and mitigate apoptosis. In TBI rats, RGFP966 reduced HDAC3 expression, increased histone acetylation, hindered neuronal loss and injury, decreased apoptosis and enhanced neurological function. These findings highlight the neuroprotective role of RGFP966 in TBI by inhibiting HDAC3 expression, although the precise underlying mechanism remains elusive.

The delicate balance between OS and AO factors is crucial in TBI-induced nerve injury and overall inflammatory responses. Nrf2, a pivotal AO transcription factor, regulates the transcription of AO genes, maintaining a balance between oxygen-free radicals and intracellular inflammatory reactions [32]. Recent evidence has suggested that the knockout of Nrf2 worsens the damage caused by TBI, while the activation of Nrf2 prevents the apoptosis of neurons and the inflammation of the brain by decreasing oxidative harm [33]. Consistently, the present study revealed that RGFP966, a specific HDAC3 inhibitor, could regulate the AO pathway by promoting Nrf2 translocation to the nucleus, which resulted in elevated expression of downstream AO factors, HO-1 and NQO-1, reducing ROS content in injured neurons and elevating essential AO enzymes to counteract ROS overproduction in the brain.

Current evidence suggests that oxidation and inflammation are intricately linked in TBI. ROS can reportedly deplete cellular AOs, induce cell membrane peroxidation and exacerbate inflammation [4, 34]. In a recent study, RGFP966 demonstrated neuroprotective effects by inhibiting inflammasome-related cytokine production in primary microglia and stroke models [35]. However, the anti-inflammatory impact of RGFP966 in TBI has not been previously reported. The NLRP3 inflammasome, implicated in different neurodegenerative diseases encompassing TBI [36], has recently emerged as a possible target for ameliorating neuroinflammation and improving cognitive and motor function in TBI [12]. Our study revealed that RGFP966 regulates NLRP3 inflammasome and downstream inflammasome pathways in the early cerebral cortex of TBI rats. However, the precise mechanisms governing NLRP3 inflammasome initiation and activation are still under exploration.

Studies suggest that the OS response and ROS after cerebral hemorrhage are potent stimulators of NLRP3 inflammasome initiation and activation [37]. Injured neurons and activated microglia release HMGB1 actively and passively into the extracellular environment, respectively [38]. HMGB1 forms a bond with TLR4, which is a part of the TLR family. This interaction triggers the Myeloid differentiation factor 88 (MyD88)-dependent pathway and contributes to direct NF-κB activation. Consequently, it stimulates the generation of proinflammatory genes and chemokines [11]. We hypothesized that attenuating OS could reduce HMGB1 release, thereby alleviating the inflammatory response. In our TBI model, the study confirmed that RGFP966 not only inhibited HMGB1 and TLR4 expression but also decreased NLRP3 inflammasome activation and the excessive release of various proinflammatory factors in the early cerebral cortex of TBI rats. Additionally, neuronal apoptosis was significantly reduced.

To validate that RGFP966 could inhibit OS, inflammatory reactions and apoptosis through Nrf2, we employed an Nrf2-shRNA lentivirus. Our findings indicated a significant reduction in the neuroprotective effect of RGFP966 after Nrf2 knockdown. Furthermore, Nrf2 knockout reversed the suppression of OS and inflammatory response induced by RGFP966 after TBI at the molecular level. It has been reported that defects in the activities of AO enzymes and exacerbated injury are present in the absence of Nrf2 [7].

There are a few possible limitations in our trials that need consideration. Initially, although previous publications have shown that RGFP966 has a protective function in regulating Nrf2 translocation into the nucleus by deacetylating HDAC3, we verified the binding ability of both through experiments such as immunoprecipitation assays. Second, our exploration of the mechanism relied solely on Nrf2-shRNA, and evidence from Nrf2 knockout animals might potentially be more persuasive.

## Conclusions

In brief, we present compelling data that RGFP966 exerts AO and anti-inflammatory effects by inhibiting the expression of HDAC3 in a neuronal OS model and a TBI rat model. The proposed molecular mechanism responsible for these positive benefits comprises the hindrance of microglia activation caused by the HMGB1/TLR4 pathway, and the inhibition of the inflammatory response mediated by the NLRP3 inflammasome by activating the Nrf2/HO-1/NQO1 AO stress signal pathway. Our experimental results may indicate a new strategy for treating brain trauma.

## Abbreviations

Abs: Antibodies; AO: Antioxidant; ARE: Antioxidant response element; ASC: Apoptotic speck-containing protein; BWC: Brain water content; Caspase1: Cysteine-requiring aspartate protease1 ; DAPI: 4′,6-Diamidino-2-phenylindole; DCFH-DA: 2′-7′-Dichlorodihydrofluorescein diacetate; ELISA: Enzyme-linked immunosorbent assay; GSH: Reduced glutathione; HDAC3: Histone deacetylase 3; HE: Hematoxylin–eosin; HMGB1: High-mobility group box 1; HO-1: Heme oxygenase-1; IF: Immunofluorescence; IL: Interleukin; MDA: Malondialdehyde; mNSS: Modified neurological severity scores; NF- κB: Nuclear factor-κB; Nrf2: Nuclear factor erythroid2-related factor 2; NQO1: NAD(P)H quinone oxidoreductase-1; OS: Oxidative stress; PVDF: Polyvinylidene fluoride; ROS: Reactive oxygen species; RT-PCR: Real-time polymerase chain reaction; SOD: Superoxide dismutase; TBI: Traumatic brain injury; TLR: Toll-like receptor; TUNEL: Terminal deoxynucleotidyl transferase dUTP nick end labeling; WB: Western blot.

## Funding

The financial support for this paper was obtained from Henan Provincial Science and Technology Research Project (No. 212102310673), Key scientific research projects of colleges and universities in Henan Province (No. 21A320056), and the Henan Province Medical Science and Technology Key Program Joint Construction Project (No. LHGJ20230779).

## Authors’ contributions

LX: Responsible for conceptualizing the project, designing and conducting experiments, collecting and analyzing data and producing the article. TA: Experimental design, experiment execution, paper writing. BJ: Data analysis and critical evaluation. QW and JS: Participation in rat experiments. JJ and JL: Participation in data analysis and interpretation. CL: Conceptualization of the study, design of experiments, provision of samples and reagents, manuscript writing and editing. The final manuscript was reviewed and authorized by all authors.

## Ethics approval

This study was accepted by the Ethics Review Committee of Zhengzhou Central Hospital (No.202263).

## Conflict of interest

None declared.

## Data availability

The corresponding author may provide the data supporting the outcomes of this research upon reasonable request.
